# Treatment of Colocutaneous Fistula in the Left Thigh

**DOI:** 10.1055/s-0039-1696728

**Published:** 2019-09-21

**Authors:** Tanweerul Huda, Bharati Pandya

**Affiliations:** 1 Department of General Surgery, All India Institute of Medical Sciences, Bhopal, Madhya Pradesh, India

**Keywords:** fistula, colonic fistula, fecal fistula, colocutaneous fistula, gastrointestinal fistulas

## Abstract

**Aim** There are few publications on the surgical management of a colocutaneous fistula in the thigh. Here, we describe a patient who presented with a 2-year history of fecal fistula in the left thigh, following a history of drainage of a psoas abscess. This is followed by a discussion of appropriate treatment modalities for this type of fistula.

**Methods** To determine the appropriate treatment for our patient with chronic fistula, we thoroughly reviewed the relevant literature in an Internet-based search and selected a staged operative approach for our patient.

**Results** Using a staged surgical procedure, we were able to resolve the colocutaneous fistula without the occurrence of comorbidities.

**Conclusion** Substantial morbidity is associated with the presence of colocutaneous fistulas. The best possible approach is prevention of its occurrence, but this is not always feasible. Measures for management of an acute fistula differ from those in patients with chronic fistula. Medical management can be more effective in acute cases, while chronic cases require surgical management. We used a staged surgical method with a few risks for our patient and he is in good health 1 year after treatment.

Colocutaneous fistulas are abnormal communications between the colon and the abdominal skin. They can occur spontaneously, as well as after an injury or a surgical procedure. Spontaneous fistulas occur mainly in patients affected by cancer, inflammatory bowel disease, diverticulitis, or appendicitis, due to the associated radiotherapy or injuries. 1 Surgical procedures performed in patients with neoplastic diseases, inflammatory bowel diseases, or adhesions often result in postoperative fistula formation. 1 Malnourishment, poor general conditions of the patient, obstruction distal to the site of development, the presence of foreign bodies, and the presence of abscesses constitute negative factors that influence the spontaneous healing of fistulas. 1 The main abdominal sources to consider in thigh abscesses are colorectal carcinoma, diverticulitis, ischiorectal abscess, appendicitis, Crohn's disease, and tuberculous psoas abscess. Etiology is typically suggested by the side of presentation: the right side is suggestive of cecal carcinoma and appendicitis, whereas the left side is suggestive of sigmoid diverticulitis and other rectal diseases. 2 The standard therapy for colocutaneous fistulas consists of conservative management, with surgery reserved for failures after maximal medical treatment, or for patients with chronic fistulas. 3

## Methods

**Fig. 1:**
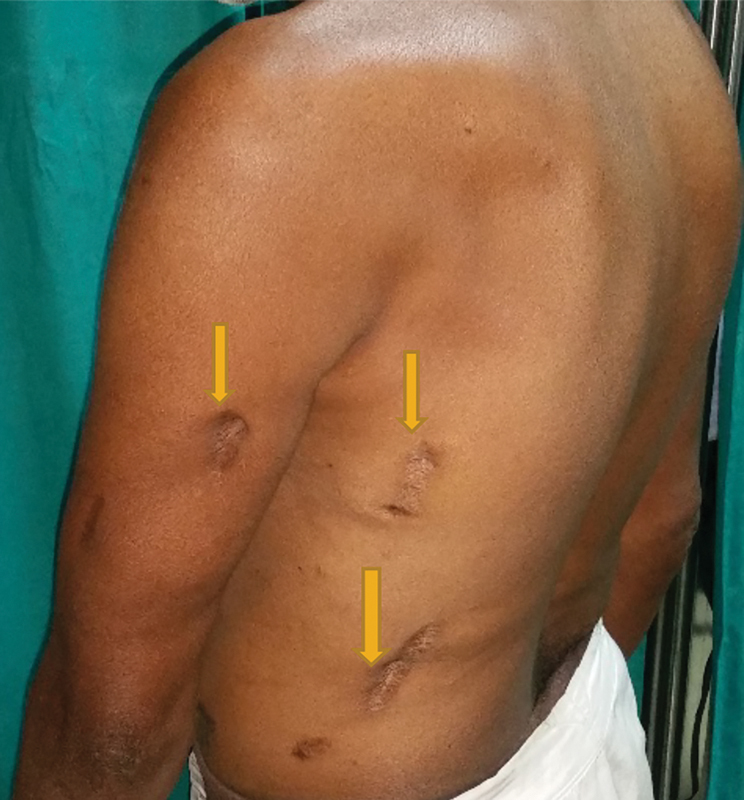
Multiple incisions over left arm and back.

A 50-year-old man presented with complaints of intermittent fecal discharge from the left thigh for 2 years and swelling in the left thigh for 2 months. Three years prior, he had developed episodes of daily fever, which was high-grade, intermittent, and associated with chills and rigor. Over a period of 1 month, he developed multiple sites of swelling on his upper limbs, back, and lower limbs, which were associated with pain and redness; thus, he was admitted to a local hospital in a critical condition. The sites of swelling were sequentially drained by multiple incisions in the arms, back, and legs over a period of 2 to 3 days ( Fig. 1 ). Pus culture revealed no bacterial growth, and was negative for tuberculosis. His wounds were healed with daily dressings and the application of broad-spectrum antibiotics. At that time, the patient reported a history of pulmonary Koch's disease, for which he had been treated 10 years prior.

**Fig. 2:**
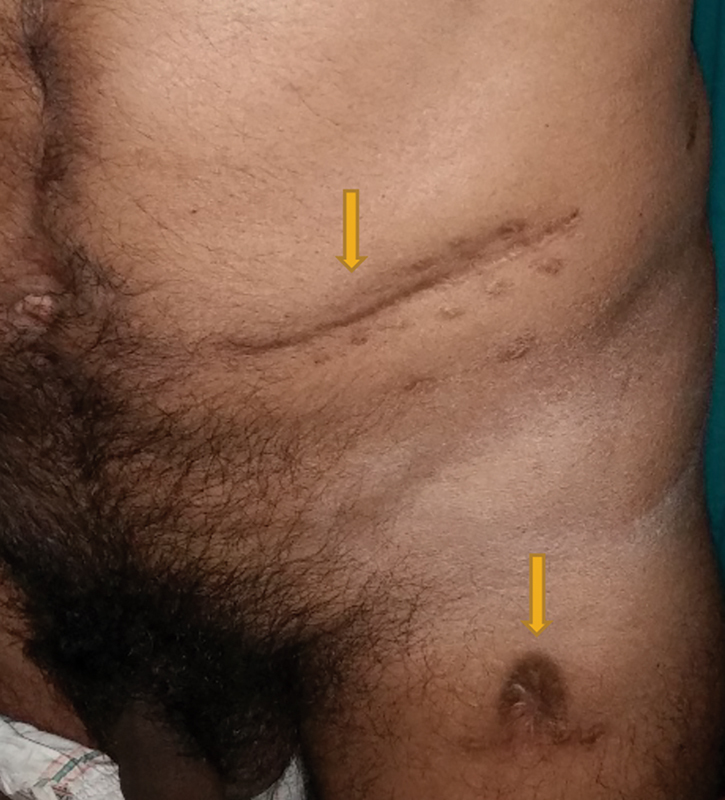
Left lower abdominal incision with left thigh fistula opening.

One year later, the patient developed painful swellings on the left lower abdomen and flanks. He exhibited an altered gait, bending more to the affected side. He was clinically diagnosed with psoas abscess, which was explored with a transverse abdominal incision; the wound was closed with a drain, and then largely healed ( Fig. 2 ). The culture was positive for tuberculosis and therefore the patient was started on antitubercular drugs and completed the full course of the treatment. The patient was reasonably healthy for the following 4 to 5 months; then, a pustule developed in the anterior aspect of the left thigh, from which a discharge emerged. This discharge was yellowish to brown in color, with gas bubbles and a feculent odor. Over the following 2 years, the wound opened and closed intermittently, discharging pus, feces, and gases from the opening; these episodes were associated with fever and left abdominal pain. The patient mentioned his history of pulmonary Koch's disease.

**Fig. 3:**
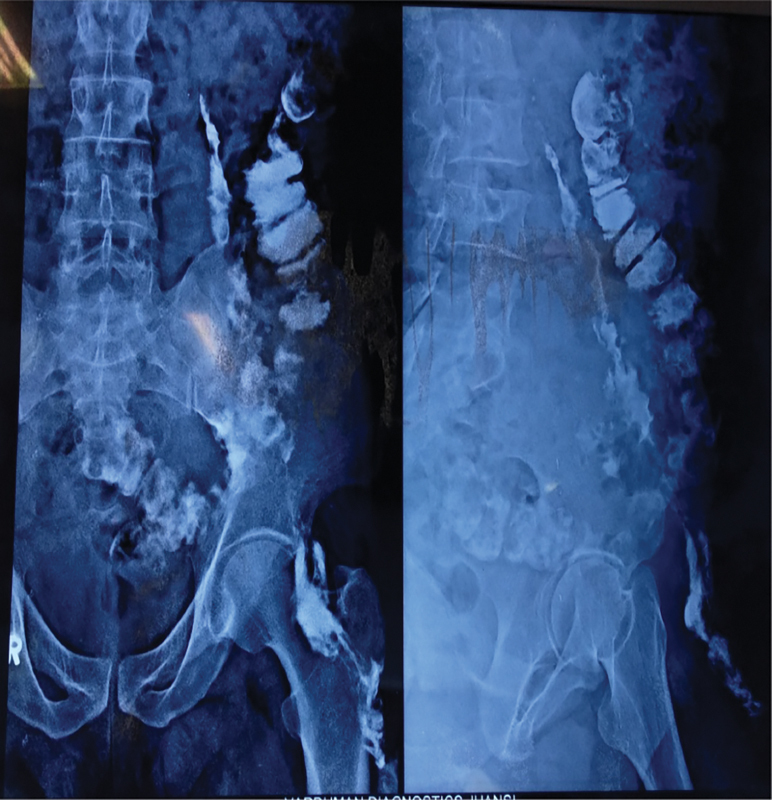
X-ray sinogram showing long tract on left side extending from its external opening in thigh up to the cavity in psoas muscle with communication to the descending colon.

**Fig. 4:**
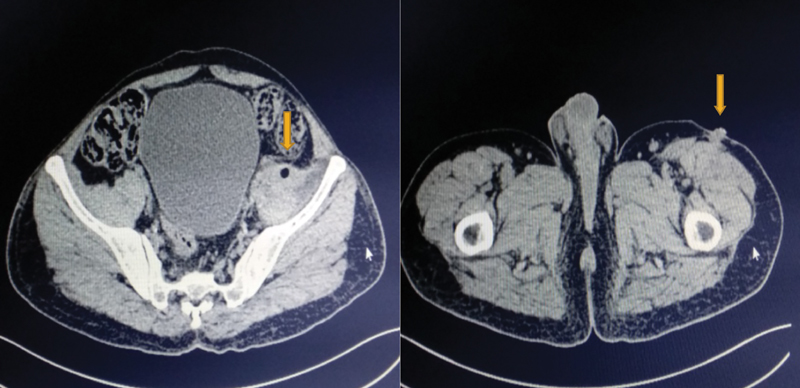
Contrast-enhanced computed tomography showing left-sided colonic fistula communicating with a cutaneous opening in thigh.

At our institute, a thorough examination was done. *X-ray sinogram* : Long tract observed on the left side, extending from the external opening in the thigh up to the cavity in the psoas muscle, with communication to the descending colon ( Fig. 3 ). *Ultrasound abdomen* : Large left retroperitoneal sinus tract (180 × 13 mm), extending up to the left proximal thigh via the left inguinal region. Few enlarged inguinal lymph nodes are present, and the largest is 18 × 7 mm. *Colonoscopy* : The colonoscope could be advanced until the terminal ileum. No internal opening of the fistula was visible, and no diverticulae were noted. *Contrast-enhanced computed tomography* (chest and abdomen): The left iliopsoas was altered and heterogeneous attenuation was present with fatty atrophy. Heterogeneous enhancement was present along the left iliopsoas muscle with multiple air-dense foci and associated fat stranding. Focal communication of the heterogeneously enhanced psoas tract was observed with the descending colon at the L4 to L5 vertebra. The features were suggestive of a fistulous communication. The left upper ureter can be observed closely abutting the psoas inflammatory tract. The tract could be observed inferiorly, extending through the left inguinal canal into the intramuscular plane of the anterior compartment of the thigh; it involved the vastus medialis and vastus lateralis, and traveled up to the skin surface ( Fig. 4 ).

**Fig. 5:**
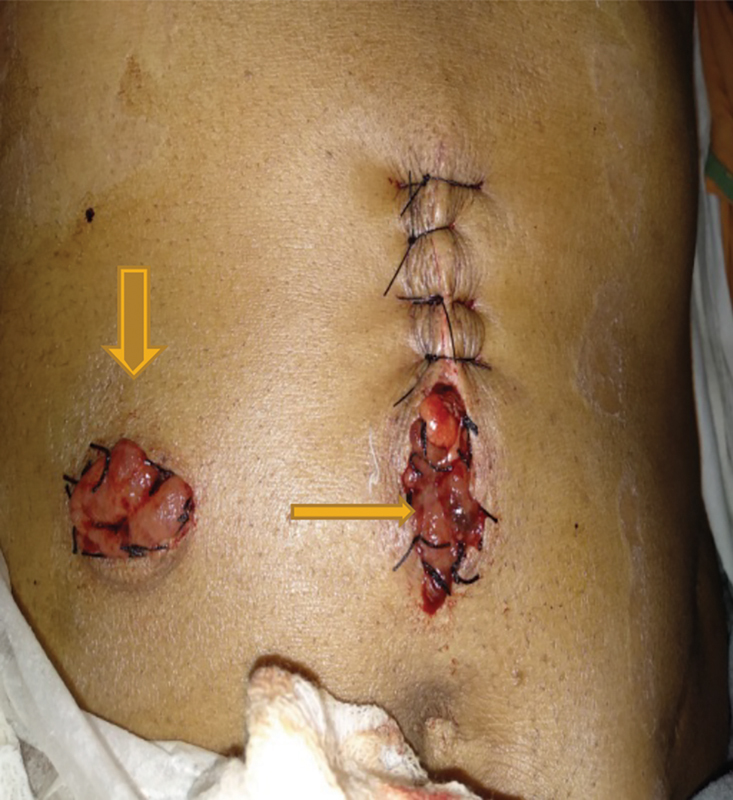
Stage 1—vertical arrow: proximal colostomy, horizontal arrow: distal loop (mucous fistula).

**Fig. 6:**
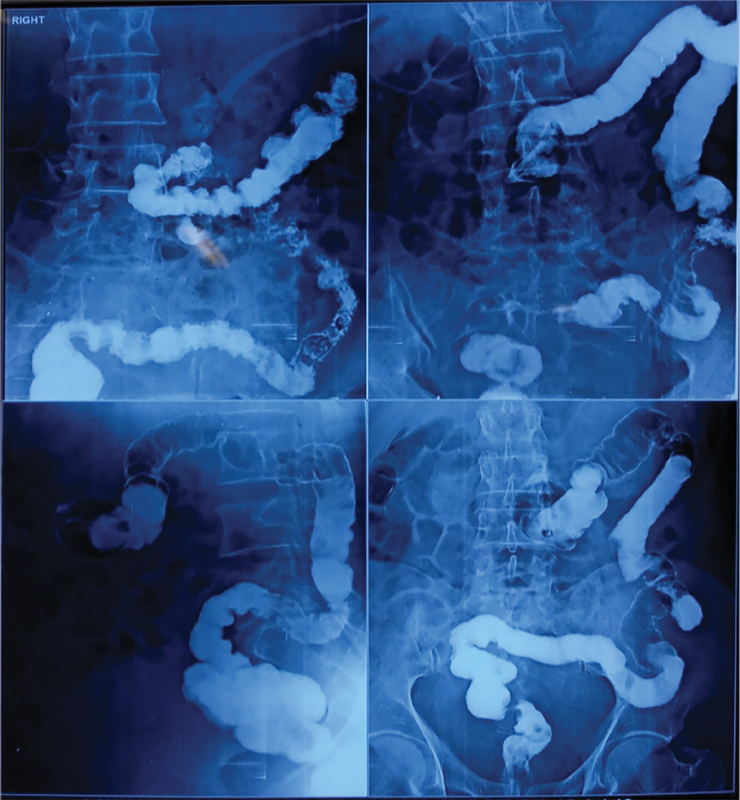
Dye study as enema showing normal anal canal. No diverticular disease. Mucosal breach seen in the descending colon with ill-defined luminal architecture.

**Fig. 7:**
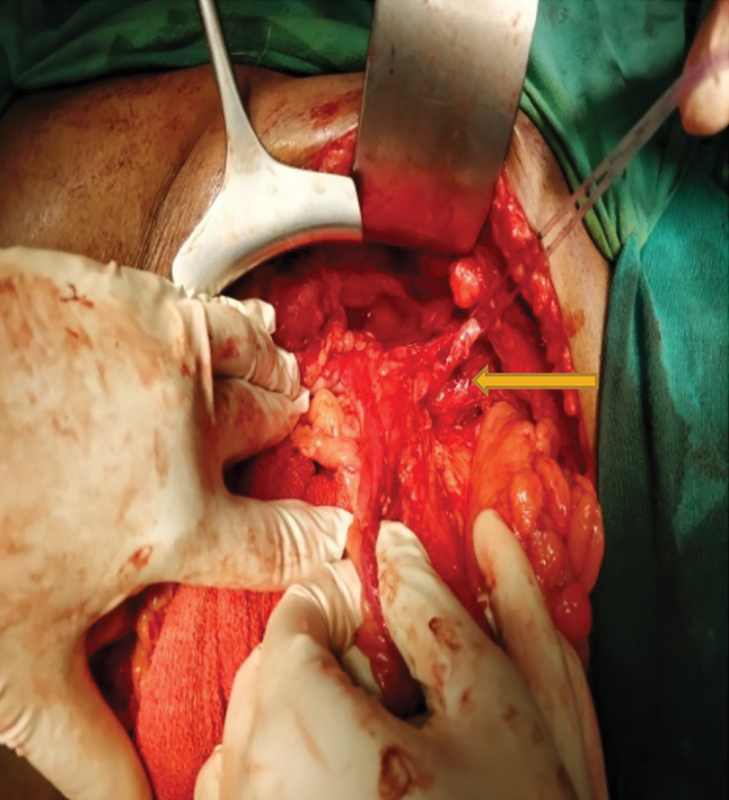
Ureter closely abutting the fistula tract.

The patient was planned for a staged surgical repair. *Stage 1* : Transverse colostomy with mucous fistula was performed under general anesthesia ( Fig. 5 **)** . *Stage 2* : The patient underwent surgical revision 8 weeks later. A preoperative ureteral stenting was done followed by a contrast enema. The dye was pushed through the distal loop and as enema. Serial radiographs were taken, which showed a normal anal canal and rectum. There was no evidence of diverticular disease. A mucosal breach was observed in the descending colon, with ill-defined luminal architecture; this was absent on colonoscopy ( Fig. 6 ). Exploratory laparotomy was performed as follows: the descending colon was identified, which was adherent to the fistulous tract; the affected lower one-third segment of descending colon was then resected along with the fistula tract and end-to-end colocolic anastomosis was performed under general anesthesia. The fistulous tract was observed closely abutting the left ureter ( Fig. 7 ). The transverse colostomy was maintained intact. *Stage 3* **:** The patient was scheduled for revision surgery 6 weeks later. A contrast enema was performed, which showed no extravasation of dye in the distal loop. The patient underwent exploratory laparotomy, and the transverse colostomy was closed by end-to-end colocolic anastomosis, thereby restoring the continuity of the bowel.

The histopathology report was suggestive of an obliterated fistula tract along with reactive lymphoid hyperplasia. No evidence of tuberculosis was noted, and no further antitubercular treatment was indicated. During the follow-up of the patient for the past 1 year, the fistula tract and external opening have completely healed.

## Results

From the time that our patient presented with colocutaneous fistula until the final outcome, he underwent a three-staged surgical approach. In the first stage, transverse colostomy with mucous fistula was performed to divert the fecal matter and facilitate drying of the fistula tract in the distal loop. In the second stage, the affected lower one-third segment of descending colon, which contained the internal opening of the fistula, was resected along with the tract; end-to-end colocolic anastomosis was performed. The transverse colostomy was maintained intact. In the third stage, the transverse colostomy was closed by end-to-end colocolic anastomosis, thereby restoring the continuity of the bowel. The histopathology report was suggestive of an obliterated fistula tract, combined with reactive lymphoid hyperplasia. No evidence of tuberculosis was noted. During the follow-up of the patient for the past 1 year, the fistula tract has completely healed, without evidence of further discharge from the external opening.

## Discussion

Fistulas are abnormal communications between two epithelialized surfaces; colocutaneous fistulas are abnormal communications between the colon and the abdominal skin. Their etiologies may vary and thus affect the selection of appropriate treatment. Spontaneous fistulas are observed in patients affected by cancer, inflammatory bowel disease, diverticulitis, and appendicitis, due to the associated radiotherapy or injuries. 1 Colocutaneous fistulas presenting alone in the thigh are rare. The main abdominal sources to consider in thigh abscesses are colorectal carcinoma, diverticulitis, ischiorectal abscess, appendicitis, Crohn's disease, and tuberculous psoas abscess.

Unusual and delayed colocutaneous fistulas have been reported in unusual circumstances, such as following rectal anastomosis, in the popliteal fossa, 4 as nephro-colocutaneous fistula, 5 as pancreatico-colocutaneous fistula, 6 and as a retained foreign body causing colocutaneous fistula. 7 A common cause remains postoperative and spontaneous occurrence secondary to diverticulitis, 1 inflammatory bowel disease, and/or malignancy. 8 Presentation in affected patients could be either delayed or acute (i.e., in the immediate postoperative period). Morbidity depends on the underlying cause. Fistulas associated with peritoneal contamination, abscesses, and malignancies carry a poorer prognosis, especially in elderly and malnourished patients. In the treatment of intestinal fistulas, medical treatment and stabilization precede attempts at surgical intervention. 9 In patients with all forms of enteric fistulas, sepsis is a major cause of mortality and must be treated aggressively. Surgical treatment is reserved for patients whose fistulas cannot be resolved with nonsurgical therapy. Favorable and unfavorable fistula sites have been described in terms of patient prognosis. 10 The esophagus, duodenal stump, pancreas, biliary tree, jejunum, and colon are considered favorable, whereas the stomach, lateral duodenum, jejunum proximal to the ligament of Trietz, and ileum are considered unfavorable. With regard to etiology, postoperative appendicular and diverticular fistulas have a better prognosis than malignant and inflammatory fistulas. Intestinal defects <1 cm and fistulas <2 cm in length also carry a favorable prognosis. Bowel segments that maintain continuation with the lumen, patients with low-output fistulas (<200 mL), and patients who undergo primary surgery in the same institution also exhibit better prognoses, compared with total disruption of anastomosis, patients with high-output fistulas (>500 mL), and patients who undergo primary surgeries in separate institutions. 10 Patients with comorbidities, immunocompromised status, presence of sepsis, malnourishment, and anemic status are known to exhibit poor healing and worse prognosis. Our patient had a chronic fistula with a long tract and a history of tuberculosis; this fistula was resolved successfully with a staged procedure that enabled him to recover from sepsis and provided a better field for subsequent surgery.

The initial management always involves hemodynamic stabilization and the provision of enteral or parenteral nutritional support, in addition to correction of sepsis. Octreotide and somatostatin have shown clear success in the early management of fistulas. 8 Management and local care of the wound are critical for the reduction in morbidity. Fistulas associated with Crohn's disease are known to benefit from anti-inflammatory agents. A short (7–10 days) course of cyclosporine has been shown to decrease fistula output, inflammation, and pain. Closure of Crohn's-associated fistulas has also been reported following the use of azathioprine and 6-mercaptopurine. Infliximab is a chimeric monoclonal antibody to tumor necrosis factor-α that has been demonstrated to heal as many as 50% of chronic intestinal fistulas in patients with Crohn's disease. 11 In patients who show no response to the conservative treatment of fistulas after a period of 6 to 12 weeks, surgical treatment should be selected, as spontaneous closure is subsequently unlikely. 2 In patients who exhibit malignancy or the presence of a foreign body, as well as distal obstruction, early intervention is indicated, even in those with low-output fistulas. 2

The surgical options available are as follows:

1. Thorough endoscopic methods using fibrin glue or staplers. 12 13
2. Laparoscopic exploration with adhesiolysis, combined with either wedge resection of the affected segment and/or stapling of the fistulous opening. Formal resection of the affected segment may be useful, combined with primary stapler or hand-sewn anastomosis of the resected bowel to restore continuity. 13 However, laparoscopic procedures carry some morbidity in terms of inadvertent enterotomies in 1 to 4% of patients, compared with < 1% in open surgeries. 10 The rates of morbidity are higher when adhesiolysis is involved: 20 to 50% of patients, compared with 13 to 19% in open surgeries.
3. Surgical procedures for enteric fistulas are ideal for chronic fistulas that have failed to heal with conservative medical management, as in our patient. However, these procedures carry significant risks. Care is needed during exploration, as well as during adhesiolysis, to minimize rough handling and inadvertent enterotomies, which can increase morbidity and recurrence rates. In open procedures, a single-staged procedure may be appropriate when the abdomen is clean and fistulas are well-consolidated. However, in patients with active ongoing inflammation at the site of the fistula, as in our patient, intermittent flaring of the abscess related to the fistula may make the entire tract adherent and friable, such that the colon may be difficult to dissect away without the development of severe procedure-related morbidity. In such cases, a staged procedure with primary diversion and subsequent resection of the segment should be used, followed by primary closure in the second stage. Furthermore, closure of the diverting colostomy could be undertaken in a third stage to avoid the possibility of friability and length-related failure of the repair.

Recurrence rates of postoperative fistulas can be minimized by using a staged approach and thoroughly preparing the patient before surgery. The risk is reported to be 18 to 33% during simple closure of the fistula 7 ; resection anastomosis carries a lower risk (18.4%), 10 and the staged procedure carries the best prognosis. Closure of the abdomen may be challenging in some patients; accordingly, de Weerd et al described the use of a sandwich-design myocutaneous flap cover to close a high-output enterocutaneous fistula (ECF). 9 Successful direct repair of an ECF has also been reported, using a surrounding fasciocutaneous flap. 10 De Vries et al found that postponing intestinal reconstructive surgery in patients with enteric fistulas led to a lower rate of recurrence, although they were unable to define the optimal timing of such surgery. 14 Some rare fistulas may close spontaneously. 12

In the postoperative period, factors that play a role in surgical outcome include proper nutrition, correction of any pre-existing anemias or hypoalbuminemia, and the management of any hemodynamic instability or electrolyte imbalances. These abnormalities should be addressed prior to operative intervention, as tissue and anastomotic healing depend on the optimization of these factors. 8 Patients who develop spontaneous fistulas due to disease require appropriate therapy (e.g., infliximab for Crohn's disease or antitubercular therapy for tuberculosis) during follow-up to prevent disease recurrence or recurrence of the ECF. 8 In patients with a malignancy-related ECF, appropriate chemotherapy and radiation should be administered to control the primary disease as needed.

## Conclusion

Colocutaneous fistulas exhibit various etiologies and courses of presentation. Appropriate management depends on the time of diagnosis. Our patient presented with a chronic and complex fistula, which required the review of available techniques for adequate management. We used a staged procedure, which is a simple and classical method, to treat our patient. The patient showed good postoperative recovery, and avoided most of the morbidities that accompany colocutaneous fistulas. Finally, we have presented an algorithm ( Table 1 ) that includes the various options available for the management of colocutaneous fistulas.

**Table 1:** Various treatment options for the management of colocutaneous fistula

A. Injury detected on operation table	B. Injury detected immediate/early postoperative period	C. Delayed postoperative period
Confirm patient is hemodynamically stable	Stabilize patient and manage infection	Stabilize patient and manage infection
Confirm and localize the site: methylene blue injection or contrast injection and C-arm study or frank feculent discharge	Investigations to confirm and localize the site: charcoal tablets for confirming, USG, fistulogram, CECT, and MRI	Investigations to confirm and localize the site: charcoal tablets for confirming, USG, fistulogram, CECT, and MRI
Primary repair	Care of the fistula site skin along with nutritional supplements and correction of anemia	Endoscopic: fibrin glue, gel foam plugs, laser therapy, and stapler closure
Resection anastomosis with or without diversion		Laparoscopic: adhesiolysis with wedge resection, segmental resection, bypass of diseased segment and/or diversion
Or exteriorizing the segment	Conservative 2–6-12 weeks trial with somatostatin analogues, Loperamide, psyllium husk, diversion colostomy or ileostomy	Open surgery: single stage or multistaged repair with adhesiolysis, segmental, or wedge resection and anastomosis and drainage of abscesses, and if necessary, diversion or bypass of the diseased segment

Abbreviations: CECT, contrast-enhanced computed tomography; MRI, magnetic resonance imaging; USG, ultrasonography.
